# Distinguishing and Biochemical Phenotype Analysis of Epilepsy Patients Using a Novel Serum Profiling Platform

**DOI:** 10.3390/brainsci10080504

**Published:** 2020-07-31

**Authors:** Jay S. Hanas, James R. S. Hocker, Christian Vannarath, Betcy Evangeline, Vasudevan Prabhakaran, Anna Oommen, James Couch, Michael Anderson, Vedantam Rajshekhar, Hélène Carabin, Douglas Drevets

**Affiliations:** 1 Department of Biochemistry, University of Oklahoma Health Sciences Center, Oklahoma City, OK 73104, USA; James-Hocker@ouhsc.edu (J.R.S.H.); Christian-Vannarath@ouhsc.edu (C.V.); 2 Department of Neurological Sciences, Christian Medical College, Vellore 632004, India; Betcyevangeline07@gmail.com (B.E.); prabhanew@gmail.com (V.P.); annasoommen@gmail.com (A.O.); rajshekhar@cmcvellore.ac.in (V.R.); 3 Department of Neurology, University of Oklahoma Health Sciences Center, Oklahoma City, OK 73104, USA; James-Couch@ouhsc.edu; 4 Department of Biostatistics and Epidemiology, University of Oklahoma Health Sciences Center, Oklahoma City, OK 73104, USA; Michael-Anderson@ouhsc.edu (M.A.); helene.carabin@umontreal.ca (H.C.); 5 Department of Pathology and Microbiology, Faculty of Veterinary Medicine, Université de Montréal, Saint-Hyacinthe, QC H3T 1J4, Canada; 6 Department of Internal Medicine, University of Oklahoma Health Sciences Center, Oklahoma City, OK 73104, USA; Douglas-Drevets@ouhsc.edu

**Keywords:** epilepsy, monitoring, serum profiling, phenotype analysis, mass spectrometry

## Abstract

Diagnosis of non-symptomatic epilepsy includes a history of two or more seizures and brain imaging to rule out structural changes like trauma, tumor, infection. Such analysis can be problematic. It is important to develop capabilities to help identify non-symptomatic epilepsy in order to better monitor and understand the condition. This understanding could lead to improved diagnostics and therapeutics. Serum mass peak profiling was performed using electrospray ionization mass spectrometry (ESI-MS). A comparison of sera mass peaks between epilepsy and control groups was performed via leave one [serum sample] out cross-validation (LOOCV). MS/MS peptide analysis was performed on serum mass peaks to compare epilepsy patient and control groups. LOOCV identified significant differences between the epilepsy patient group and control group (*p* = 10^−22^). This value became non-significant (*p* = 0.10) when the samples were randomly allocated between the groups and reanalyzed by LOOCV. LOOCV was thus able to distinguish a non-symptomatic epilepsy patient group from a control group based on physiological differences and underlying phenotype. MS/MS was able to identify potential peptide/protein changes involved in this epilepsy versus control comparison, with 70% of the top 100 proteins indicating overall neurologic function. Specifically, peptide/protein sera changes suggested neuro-inflammatory, seizure, ion-channel, synapse, and autoimmune pathways changing between epilepsy patients and controls.

## 1. Introduction

Epilepsies are brain disorders characterized by recurrent seizures coupled with reduced thresholds for such seizures [1,2]. Approximately 3 percent of the world’s population will experience seizures that can be interpreted as epilepsy in their lifetime [3,4]. A practical clinical definition of epilepsy is having at least two seizure episodes separated in time and within a ten year period [5]. Approximately 60% of epilepsy patients suffer from symptomatic or structural epilepsy, caused by evident brain changes such as stroke, traumatic brain injury (TBI), microbial infection [6,7,8]. In about 40% of epilepsy cases, symptomatic/structural brain changes are not evident (non-symptomatic, idiopathic, epilepsy of unknown etiology) upon MRI or CT brain imaging. A much lower percentage of epilepsies appear to be caused by single gene mutations, for example in ion-channel proteins, which become evident in infancy [9]. Unknown gene changes in epilepsy in general could be very complex since 50% of the genes in the human genome are expressed in the human fetal developing brain [9]. Unknown environmental inputs contributing to epilepsy in context with such gene changes are also likely complex. Anti-epileptic drugs (AEDs) do suppress recurrent seizures in a majority of cases; however, about 30–40% of affected individuals remain refractory to drugs. Significantly, these medications do not affect or ameliorate underlying epileptogenesis biochemical/cellular processes [2].

Abilities to monitor and understand epileptogenesis and its biochemical/cellular mechanisms are lacking [10]. It would be important to develop accurate and robust non-invasive/less-invasive monitoring capabilities, for example involving peripheral blood biomarkers, to further understand underlying biochemical mechanisms and to improve therapeutic approaches. Inflammation-related sera proteins are proposed to be biomarkers for monitoring epileptogenesis and also to glean therapeutic targets [11]. Inflammatory cytokine mediator IL-6 levels are increased in epileptic patients when compared to controls [11]. Creatine kinase (CK) is elevated in the blood of patients experiencing epileptic seizures versus those with non-epileptic seizures [11,12]. CK was shown to have a test metric sensitivity (true positive rate) of 75%, which is likely too low for generalized diagnostic use.

The purpose of the present studies is to examine whether a novel methodology can aid in the identification and monitoring of patients with non-symptomatic epilepsy. This method also lends itself to gleaning underlying biochemical mechanisms as well as identifying potential novel biomarkers and therapeutic targets. The methodology involves an all-liquid mass spectrometry (MS) platform using unfractionated serum to distinguish and monitor different disease states. This MS approach was successful in identifying patients with epilepsy caused by brain infection, traumatic brain injury TBI, and early-stage cancers [8,13,14,15].

The disease specific monitoring ability and discriminating ability of this MS platform may likely be due to the large number of distinguishing components analyzed at the same time using an innovative mass peak analysis procedure [13,14,16]. The more differing components so analyzed, the greater the disease discrimination/monitoring powers of a biomarker platform. The major hypothesis of this approach is that disease conditions such as epilepsy elicit disease-specific and systemic biochemical responses from organs and tissues [17,18]. Disease-specific biomolecules will be shed or secreted into the peripheral blood and are observable and distinguishable with this electro-spray ionization mass spectrometry (ESI-MS) profiling platform [13,14]. These physiological and phenotype changes can take the form of stress/defense/homeostatic and other signaling responses, and direct inputs from disease tissues [17,18].

Peptides/proteins identified may distinguish epilepsy patients from control individuals and could help identify disease mechanisms, potential therapeutic targets or novel biomarkers. The ability of this platform to distinguish/monitor sera of patients with epilepsy, based on their respective serum biomolecule mass peak profiles, is demonstrated for the patient groups examined in this study. The bioinformatics/systems biology analysis of peptide/protein changes associated with epilepsy suggest cell pathways/biochemical systems affected/altered include neuro-inflammation, seizure/epilepsy, synaptic transmission, cognitive impairment, behavior, TBI, brain damage, blood–brain barrier, and ion-channels. Autoimmune pathway connections were also observed, lending support to a previous association with autoimmunity and epilepsy [17,18,19].

## 2. Materials and Methods

### 2.1. Study Participants

This study was approved by the Human Subjects Institutional Review Boards of CMC Vellore and of the University of Oklahoma Health Sciences Center, Oklahoma, USA. All subjects gave their informed consent for inclusion before they participated in the study. The study was conducted in accordance with the Declaration of Helsinki, and the protocol was approved by the University of Oklahoma Health Sciences Center Institutional Review Board (IRB, Project identification code #16125). Study participants sought care at the Christian Medical College (CMC) and Hospital, Vellore, India between January 2013 and October 2014 for seizure or headache issues. Written informed consents were obtained from study participants prior to blood specimen retrieval and any treatments. Seizure patients had at least two seizures in the last ten years and at least one seizure in the previous 7 months, indicating an epilepsy disorder [5] Seizure patients (*N* = 29) were found to have no evidence of parasite or tumor or other structural brain lesions on MRI (*N* = 21) or CT (*N* = 8) imaging, and were seronegative for antigens and antibodies to the larval stages of *T. solium* and *T. saginata*. These seizure patients (*N* = 29) were diagnosed as having non-symptomatic epilepsy of unknown etiology by the CMC Clinical Staff.

The mean age of the epilepsy patients was 27 with 65% being males (Table 1). The mean age of the control subjects was 34 and 59% were female (Table 1). Their brain imaging was normal with no history of seizures, brain tumor, head trauma, HIV (human immunodeficiency virus), HBV (hepatitis B virus), HCV (hepatitis C virus). This group was designated as presenting with idiopathic headaches (Table 1). All MRI and CT images were read by one of the authors (V.R.). Patients had not taken anti-inflammatory drugs at least 7 days prior to enrollment, and were not visibly ill at the time of blood collection. Sera were obtained from peripheral blood at the CMC Hospital, Vellore, according to standard procedures [20]. Aliquots (250 µL) were frozen at −80 °C, and not reused after initial freezing and thawing. Other socio-demographic and clinical characteristics are summarized in Table 1.

### 2.2. Direct Electrospray Mass Spectrometry (ESI-MS) of Sera from Patients With Epilepsy and Control Subjects

An LCQ ADVANTAGE ion-trap electrospray MS instrument (ThermoFisher), was used for obtaining serum MS spectra and for tandem MS/MS peptide/protein identifications. The spectral data were analysed as described previously [8,14]. Briefly, three high-resolution mass spectra were obtained from each serum sample over a mass divided by charge (m/z) range of 400 to 2000. Spectral data were recorded and extracted using the manufacturer’s software (Qual Browser: version 1.4SR1), and normalized to a sum value of 100 intensity units in non-overlapping segments of 10 m/z. Mass peak areas were recorded/transformed into centroid m/z peak area values (valley to valley) using Mariner Data Explorer 4.0.0.1 software (Applied BioSystems). All serum samples were processed in an identical fashion. The same serum samples (epilepsy or control) were also analysed on a lower resolution compact desk-top single quadrupole ESI-MS instrument (Expression CMS, Advion, Inc., Ithaca, NY, USA) as described previously [8].

For tandem MS/MS peptide/protein identifications, ions encompassing the range of 900–1008 m/z were analysed in 10 epilepsy patient and 10 control individual sera samples that were age and sex matched as best as possible. This m/z range was chosen based on empirically determined optimal machine performance for MS/MS analysis of unfractionated serum samples. A 35% ionization energy was utilized on each m/z unit parent ion and fragmentation was observed for 5 min. The MS/MS signal data analysis utilized ThermoFisher Proteome Discoverer 1.0 SP1 with a human and *T*. *solium* non-redundant databases as downloaded from the National Center for Biotechnology Information (NCBI). On average, serum samples contained 1.95 (range: 0–5) parent ions with significant differences, as determined by standard deviation of MS spectral data, between the pre and post MS/MS scans. MS/MS search settings were applied [enzyme name = no-enzyme/ no digest]: precursor mass tolerance = 1.8 Da, fragment mass tolerance = 0.8 Da, “b” and “y” ions were scored, and any dynamic modifications for oxidation (C and M amino acids), phosphorylation (S, T, and Y), methylation (C), were noted with a maximum of 4 modifications per peptide. Protein identifications required a minimum of two unique peptides and a cross-correlation range (Xcorr) ≥ 1.7, in line with previous studies [13,14]. The identified sequences were also searched using the National Center for Biotechnology Information NCBI online search database Basic Local Alignment Search Tool (BLAST). A “hit” in the protein database search is scored for each MS/MS scan when the Xcorr, identifying a peptide sequence, is higher than or equal to 1.7. Each sample was scanned multiple times for a total of 5 min duration at each m/z. Each identification of a peptide/protein sequence was termed a “hit”. The number of patient sera samples out of ten indicating the presence of the same peptide/protein is reported. Identified protein names and the number of Identified MS/MS sequence spectra identified “hits” were imported each as log(base 2) ratios of epilepsy/control for Ingenuity Pathway Analysis (IPA, QIAGEN) [23]. Proteins have been inspected for protein function using the Medline and PubMed online databases.

### 2.3. Statistical Analysis

**Figure 1:**
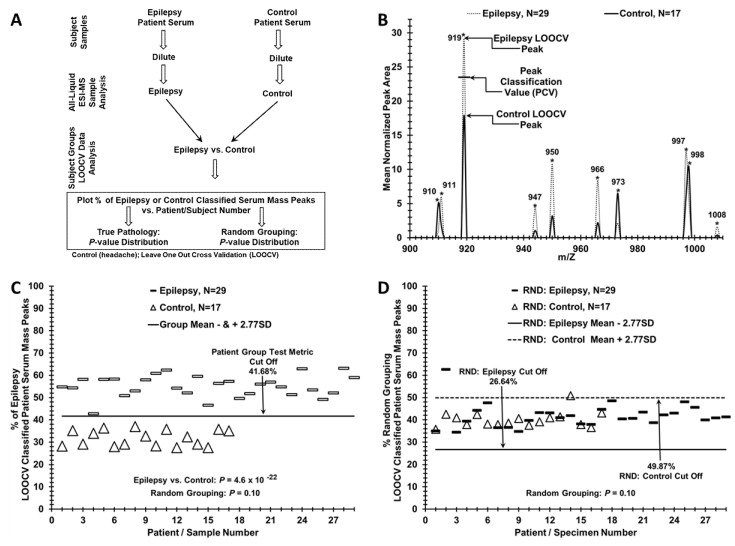
Distinguishing Epilepsy vs. Control patients using leave one [serum sample] out cross-validation/ peak classification value (LOOCV/PCV) serum mass peak analysis. (**A**) Flow chart outlining the serum sample handling and mass spectrometry processing of the binary patient/subject group analysis. (**B**) Serum mass peak Scoring of the LOOCV/PCV (leave (one serum sample) out cross-validation/peak classification value) procedure used to classify mass peaks either as “epilepsy” or as “control” for a “left out” sample, (a limited sample range 900–1008 m/z is displayed) of significant group discriminatory mass peaks. The PCV example for the peak at 919 is exhibited and used to classify “left out” peaks as either “epilepsy” (peak area above this PCV) or control (peak area at or below this PCV). (**C**) Serum discrimination of epilepsy patients (dashes) from controls (triangles) by a % of LOOCV classified mass peaks. A cut off value is indicated (− or + SDs from the seizure or control groups respectively) to determine test metric values (e.g., true positives). (**D**) A lack of serum sample discrimination is demonstrated which results when the two different sample groups are mixed together randomly followed by the same LOOCV mass peak analysis.

Leave one [serum sample] out cross-validation (LOOCV) involving a novel peak classification value (PCV) procedure was used to help distinguish serum samples of epilepsy patients from control individuals (Figure 1) [8,14]. Triplicate averaged mass peak areas at individual m/z mass peaks from diluted sera were compared for significance between the epilepsy patients and controls using one-tailed Student’s *t*-tests and used in the LOOCV analysis (Figure 1A). One sera sample and its mass peaks from either the control or epilepsy groups is alternatively “left out” to build a series of unique N–1 LOOCV “left in” significant (*p* < 0.05) mass peak area “difference” datasets. The mass peaks of each “left out” sample were then compared to all the “left in” mass peaks in their unique N-1 LOOCV dataset. This comparison involves the use of a peak classification value (PCV) used to classify each “left out” sample at each significant “left in” peak of each N-1 LOOCV dataset. Whether a “left out” peak area falls above or below the PCV determines if it should be classified into the epilepsy or control group. For example, peak 919 in Figure 1B is classified as an “epilepsy” peak in the “left in” database because the higher mean area is assigned to this group (dash). If the 919 peak from the “left out” sample has a peak area above the PCV then it is classified as an “epilepsy” peak. If it falls below or equal to this PCV, then the “left out” peak is classified as a “control” peak. Peak classifications are performed for all “left out” peaks in all “left out” serum samples against their respective N-1 “left in” LOOCV mass peak databases resulting in a summed % total LOOCV peak sera score (patient score). This procedure results in sera samples displaying a combination of “epilepsy-peaks” and “control-peaks”. This % of total mass peaks classified as epilepsy for the left-in dataset is assigned each “left out” sample and plotted on the y-axis versus the individual serum samples on the x-axis in Figure 1C. To check for over-fitting [24,25,26] of the large datasets, sera samples were randomized to the control or epilepsy group (Figure 1D) using the RND (randomization) function in Excel and manually balanced to retain gender and age ratios of the initial groups [8,14]. Upon randomization, LOOCV was performed again exactly as described above. Cohen’s-*d* values were calculated from the %LOOCV means and standard deviations to approximate the discriminatory effect size between two groups [27]. Cohen’s-*d* serves as a measure of statistical power (ability to detect type II errors-false negatives), and is estimated for given sample sizes as described [27,28].

### 2.4. Test Metrics

**Figure 2:**
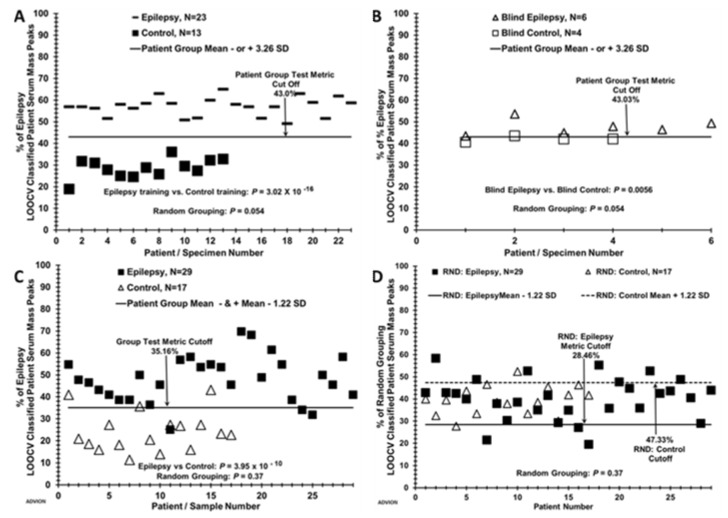
Distinguishing blinded “left out” sera samples, and LOOCV with a lower cost desk top mass spectrometer. (**A**) Six blinded epilepsy and 4 control samples from each group were removed from the database and the remaining samples (*N* = 23 for epilepsy and 13 for control) were used as a training set. A % LOOCV classified mass peak group test metric cut-off of 43.0% was established 3.26 SD’s from each respective group mean as described in the methods. (**B**) Six blinded epilepsy samples and 4 control samples were LOOCV tested against the test metric cut off of 43.0% determined by the training group dataset. (**C**) Epilepsy patient and control % scores for the LOOCV dataset (*N* = 29 epilepsy and 17 control) obtained using a small-footprint, lower ion-resolution, and limited m/z range (200–1200) MS instrument (Advion, Inc.). Group test metric cut off is 35.16% with 1.22 SD. (**D**) Random LOOCV grouping of epilepsy patients and controls from panel C demonstrates lack of group separation (*p* value 0.37). A lower resolution and lower cost MS instrument than the LCQ Advantage (Expression CMS ESI-MS instrument, Advion, Inc.) performed reasonably well in distinguishing subjects of the epilepsy group (*N* = 29) as different from the control individual (*N* = 17) group (Figure 2, panels C,D). The mass analyzers in these two instruments have differing physics and electronics (ion trap vs. single quadrupole), and the group discriminatory *p*-value is larger for the CMS instrument (10^−10^) than the LCQ instrument (10^−22^). Whereas the LCQ had no false positives or false negatives, the CMS instrument resulted in three false positives and three false negatives, Figure 2C. The randomization *p*-value is higher with the CMS instrument vs. the LCQ instrument (0.37 vs. 0.10). These results do suggest a less accurate instrument with reduced m/z range can still detect enough mass spectral signal differences between these two groups. This ability strengthens the conclusions that the biomolecules observable in the serum and differing among the study groups could possibly help in the diagnosis and monitoring epilepsy.

**Figure 3:**
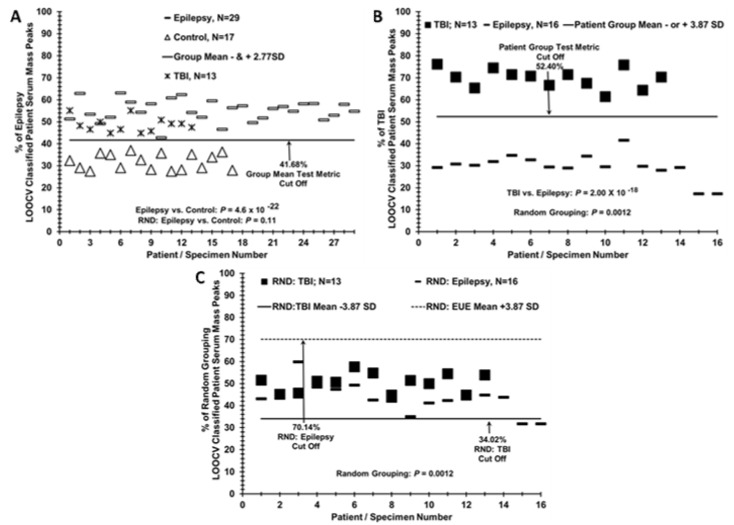
Sera from TBI patients are more closely related to Epilepsy patients when compared to controls. (**A**) Segregation of TBI patient sera (Xs) with epilepsy sera (dashes) when both are LOOCV/PCV compared to control individual sera (triangles). (**B**) Distinguishing TBI patient sera from epilepsy patient sera when compared to each other directly by the LOOCV analysis. (**C**) Random LOOCV grouping of sera from epilepsy patients and TBI patients from panel B; demonstration of lack of group separation (*p* value 0.0012).

A group test metric “cut off” was calculated from the mean % LOOCV classified peaks from each group minus (epilepsy), or plus (control), an equivalent number of standard deviations (SD) exhibited, e.g., in Figure 1C and as previously described [8,14]. LOOCV cut offs were used to determine False Positives (FP), True Positive (TP), False Negative (FN), and True Negative (TN) values for classifying controls and epilepsy patients into the proper group (Figure 1, Figure 2 and Figure 3). Sensitivity is the percentage of epilepsy patients classified as epilepsy because their % of total LOOCV was above the epilepsy LOOCV threshold cut off. The specificity is the percentage of control patients classified as control because their % of total LOOCV was at or below the epilepsy LOOCV cut off.

## 3. Results

### 3.1. Distinguishing Sera of a Patient Group with Epilepsy from a Control Individual Group Using LOOCV/PCV ESI-MS

Distinguishing a patient group with epilepsy from a control group with this ESI-MS serum profiling platform is illustrated in Figure 1C. Patient scores (y axis) for the % of the total LOOCV classified serum mass peak dataset were used to compare epilepsy patients (dashes) and control individuals (open triangles, x axis). A clear demarcation/separation is observed between the epilepsy patients and control patients in the % of epilepsy LOOCV classified patient serum mass peaks (y axis, 106–118 peaks utilized). The cut off of 41.68% epilepsy LOOCV classified mass peaks yielded a strong separation of the epilepsy group from the control group as evidenced by the very low *p*-value (4.6 × 10^−22^). The “Group Mean Test Metric Cut Off” represents the combined cut off values of both epilepsy and control groups and is set at equidistant differences of 2.77 SD’s from each respective group mean (–2.77 SD-standard deviations higher scoring group) or (+2.77 SD lower scoring group). These narrow standard deviations indicate considerable homogeneity in both of these groups despite their somewhat uneven nature in terms of sample numbers between the groups and a gender bias toward males within the groups (Table 1). An individual patient serum score > “Cut Off” value suggests higher identity to the epilepsy group. Whereas, a score ≤ “Cut Off” value suggests higher identity to the control group. The cut off in Panel C indicates there are no false positives or false negatives in this separation. The epilepsy vs. control comparison was performed previously with sera samples from patients recovered from neurocysticercosis (RNCC) epilepsy [8]. Sera mass peak groupings were different in the present Figure 1C, and this led to improved group *p*-value separation from 10^−18^ [8] to the present 10^−22^. When sera samples from these study subjects are randomized between the two groups and the same LOOCV/PCV process is repeated, nearly all samples are LOOCV classified as members of both the control and epilepsy groups with no separation, as exhibited in Figure 1D. This random grouping of patient serum samples demonstrates a lack of separation between patient groups. Specific group cut-off lines are shown and now represent different values, due to random patient grouping, even though the calculation methodology did not change. The lack of difference between the randomized groups is evidenced by a much larger *p*-value obtained (*p* = 0.10). These results are consistent with an expectation of minimal over-fitting and the potential presence of a physiological basis being responsible for the discrimination observed between these groups.

### 3.2. Distinguishing Blinded Epilepsy Sera Samples, and LOOCV with a Low-Cost Desk Top Mass Spectrometer

A blinded sample experiment was performed by randomly removing six epilepsy samples and four control samples from the dataset used in Figure 1C, and re-run the LOOCV using the remaining 23 epilepsy and 13 control samples as a “training set” in Figure 2A. The training set distinguished samples from the two study groups with a *p*-value of 3.02 × 10^−16^; randomization resulted in a *p*-value of 0.054, suggesting minimal over-fitting in the training set. The 10 “blinded” samples were then classified using the epilepsy LOOCV test metric cut off determined by the training set (43%) and displayed in Figure 2B. A total of 9 out of 10 samples were correctly classified; one blind sample from the control group was above the cut off value of the epilepsy training set so would have mistakenly been classified as false-positive “epilepsy”. The analysis of additional samples in a future study could conceivably improve such blind sample discrimination.

### 3.3. Epilepsy Patient Sera Segregates from TBI Patient Sera when Compared to Controls but Segregates from TBI Patient Sera when Compared Directly

Figure 3A exhibits where the “left out” sera mass peaks from 13 male United States Veteran patients (Xs) who suffered a mild (loss of consciousness 1–30 min) TBI (male age mean = 39.9) group-classify using the LOOCV/PCV procedure employed in the Figure 1C separation of epilepsy patients from controls [14]. All 13 sera (Xs) segregate with the epilepsy samples (dashes) versus the control samples (triangles), which is consistent with some physiological similarity between the two brain disorders when compared to control individuals with minor headaches. Importantly, this LOOCV/PCV procedure is able to distinguish the sera of these TBI patients (dark squares) from the epilepsy patients (dashes) from these TBI-patients with a low *p* value (10^−18^ panel B). The RND-discrimination *p*-value is more significant for the epilepsy vs. TBI discrimination (0.0012, panel C) than for the epilepsy vs. control (0.10, Figure 1C). Despite this significant *p*-value for the randomization of the TBI and epilepsy patient samples, little if any group separation is observed (panel C).

### 3.4. Test Metric Data for Epilepsy, Control, and TBI Serum LOOCV Profiling Comparisons

Table 2 summarizes the test metrics for the LOOCV data in Figure 1, Figure 2 and Figure 3. Patient groups tested are listed in the far-left column. % LOOCV classified mass peak mean values with standard deviations (SD) for the two comparative groups are given in panel I. The performance of ESI-MS to classify subjects into their true study group was high, with a sensitivity and a specificity of 100% and 97%, when the full LOOCV dataset was used for the epilepsy vs. control comparison (panel II). Cohen’s-d effect size values are provided in panel II. These values are calculated from the % LOOCV means and SDs to obtain a sense of the effect size which is a measure of the size of the observed differences between the two groups under comparison [27]. Cohen’s-d is an indirect measure of statistical power (ability to detect type II errors-false negatives). The large Cohen’s-d values here have an estimated power of >0.90 and bolster the reliability of the sample sizes utilized here [27,28]. Test metrics using the lower resolution, lower m/z range, single quadrupole desk-top ESI mass spectrometer are exhibited in panels I and II. All metrics are diminished when compared to the LCQ instrument.

### 3.5. Phenotype Assessment of Epilepsy Patients versus Control Individuals Using TANDEM MS/MS of Serum Peptide/Proteins, and Bioinformatics Cell Pathway/Disease Mechanism Analysis

MS/MS analysis of 10 epilepsy patient sera and 10 control sera, in a range between 900 and 1008 m/z, was employed to examine peptide/protein differences between these two sera groups. This 900–1008 range was empirically shown previously to provide ample ionization for serum MS/MS peptide identification [14]. To focus on peptides/proteins with larger differences between the two groups, a subset of the top 100 peptides/proteins showing at least a two-fold difference in number of positive sera between epilepsy and control groups was chosen and is exhibited in Table 3 and Table 4. A MS/MS “hit” (single peptide identification) ratio between the two groups of at least a rounded off value of 1.5 was also employed. A total of 182 peptides/proteins meet these criteria and are exhibited as Supplemental Table S1. A PubMed/Medline search of these 100 differentially expressed peptides/proteins in Table 3 and Table 4 showed 70% were related to neurological function, 45% to immune/inflammation, 32% to seizures/epilepsy, 24% to ion-channels, and 10% to blood–brain barrier (BBB). Seizure/epilepsy related proteins (shaded cells in Table 3 and Table 4) include: SLIT2 (Slit guidance ligand 2 [29]), EPHB2 (EphrinB receptor 2, [30]), LAMA2 (Laminin alpha-2, [31]), ADAM11 (ADAM (A disintegrin and metalloprotease domain 11, [32]), P2RX7 (Purinergic receptor P2X 7, [33]), SLC4A4 (Solute carrier family 4 member 4, [34]), KCNQ2 (Potassium Voltage-Gated Channel Subfamily Q Member 2, [9]), VWF (von Willebrand factor, [35]), RYR2 (Ryanodine receptor 2, [36]), RECK (Reversion Inducing Cysteine Rich Protein With Kazal Motifs, [37]), ATP7B (ATPase Copper Transporting Beta, [38]), NF1 (Neurofibromin 1, [39]), HDC (Histidine Decarboxylase, [40]). Two other important seizure/epilepsy related proteins in Table 3 and Table 4 are CACNA2D2 (Calcium Voltage-Gated Channel Auxiliary Subunit Alpha2 delta 2, [41]) and ASTN2 (Astrotactin 2, [42]). Both these proteins have ion-channel functions which have important roles in epileptogenesis [9]. It is noted that 16 out of 32 seizure/epilepsy related peptides/proteins in Table 3 (50%) have ion-channel relatedness.

**Figure 4:**
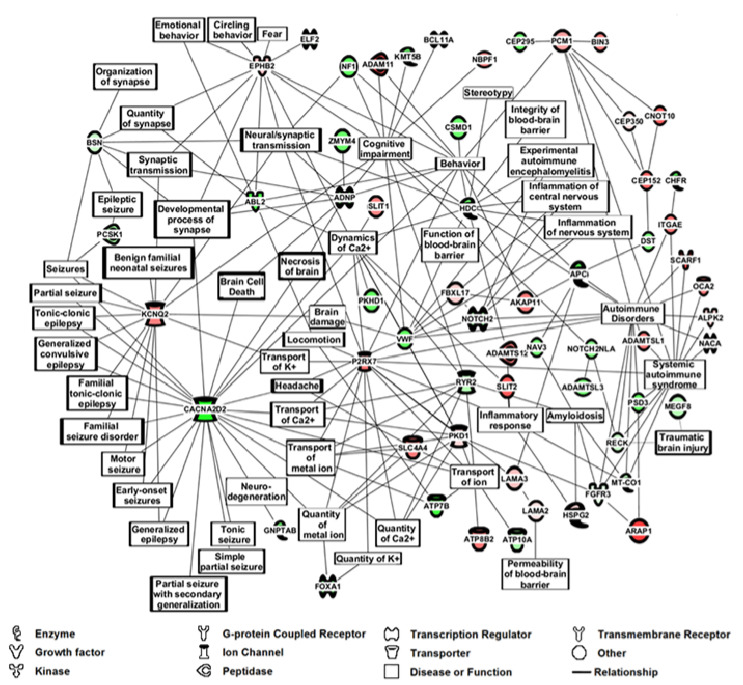
IPA m/z Range MS/MS Serum Data Analysis for Epilepsy Patients vs. Controls using top 100 Peptide/Proteins from Table 3 and Table 4. Ingenuity Pathway Analysis (IPA), (Qiagen, Inc.) of the 100 peptides/proteins exhibited in Table 3 and Table 4 having a 2× difference in positive sera number between epilepsy and controls, and a 1.5× difference in MS/MS “hit” (single peptide identification) ratio between the two groups. The protein function legend is at the bottom of the figure.

The proteins/peptides expressed differently between these epilepsy and control groups were analyzed by Ingenuity Pathway Analysis (IPA) to potentially identify affected networks of cellular/biochemical/disease pathways/systems (Figure 4, 60 out of 100 proteins present in Table 3 and Table 4). IPA is used in this context to predict cellular pathways that are changing based on altered gene expression parameters [23], in this case, serome peptidome changes which are valid gene expression markers for disease states [43]. Pathways affected and major proteins present in such pathways include neuroinflammation (HDC, NOTCH, VWF), synapses and synaptic transmission (BSN, EPHB2), cognitive impairment (SLIT2, RYE2, NF1), behavior (PCM1, EPHB2), seizure/epilepsy (KCNQ2, CACNA2D2), blood–brain barrier (HDC and VWF), brain damage (EPHB2, VWF, CACNA2D2), brain cell death (ADNP and CACNA2D2), traumatic brain injury (TBI, RECK and MEGF8), and ion-channels (KCNQ2, CACNA2D2), and autoimmunity (VWF, RECK, SCARF1). IPA of the top 182 proteins in Table S1 with autoimmune emphasis is exhibited in Figure S1 (74 proteins present).

A line connection between two proteins or between a protein and a “hub” in Figure 4 (hub defined as a biological function or disease), for example, between ABL2 and EPHB2 or between EPHB2 and the hub synaptic transmission, represents published biological findings compiled in IPA software. Of note, some of the proteins listed in Table 3, Table 4 and Figure 4 may not have a direct connection to epilepsy, but serve as connections to other biomolecular pathways of possible interest like locomotion and amyloidosis. Finding proteins/peptides known to be related to seizure/epilepsy phenotypes in an epilepsy patient comparison with control individuals in Table 3, Table 4 and Figure 4 provides initial support for the ability of this mass profiling platform to assist as a potential monitoring platform and push forward to decipher the epilepsy pathology and phenotype. Future studies will examine larger numbers of serum samples in these contexts, and also test for peptide/protein presence in sera using immunoassays. Such analyses are complex since mostly peptides and not intact proteins are being identified by MS/MS. Matching available antibodies and their epitopes to peptides is a complex process. In some cases, this will likely involve de novo acquisitions of peptide-specific antibodies.

The authors recognize the imbalance of male and female participants but were unable to demonstrate any of the presented results specifically associated with patient gender.

## 4. Discussion

Biomarkers are excellent tools for monitoring and understanding diseases such as epileptogenesis and seizure as well as aiding in treatment [10]. Although biomarker progress on epilepsies has been slow, recent advances in large input/throughput approaches (genomic, transcriptomic, proteomic, metabolomic) show promise [10]. It is important in this “omic” context to examine cellular/pathophysiological networks possibly having roles as these diseases are complex. Developing such biomarker and cell network approaches using readily available bodily sources such as peripheral blood would be helpful. The present studies purpose was to examine a novel methodology to see whether it could help identify and monitor patients with epilepsy, in this case of unknown etiology. The MS method uses unfractionated serum to help distinguish and monitor epilepsy patients from control individuals. The hypothesis of this approach is that epilepsy induces organs and tissues to release/shed specific biomolecules into the peripheral blood involved in the disease state as well as in specific systemic responses to that disease state. Examination of biomolecules in peripheral blood, e.g., peptides/proteins that change with epilepsy, has the potential to provide diagnostic, phenotypic, mechanistic, and therapeutic insights into this disorder. The serome peptidome is a valid gene expression entity for study and was shown to correlate with specific disease states [14,43]. This study utilized serum samples gathered from patients presenting with a history of seizure (seizure group) and patients presenting with headache (control group) and sought care at the Christian Medical College (CMC) and Hospital, Vellore, India between January 2013 and October 2014 for either seizure or headache issues. Alternative explanations, as provided by standard of care, for seizure were excluded as described in the Materials and Methods.

MS profiling of sera identified mass peaks changing significantly upon comparison of patients with epilepsy versus controls individuals who sought treatment for idiopathic headaches but with no brain lesions upon examination. Randomization of serum samples between these two groups followed by LOOCV mass peak analysis resulted in loss of group-specific discrimination ability, suggesting a physiological basis for the epilepsy vs. control discrimination. These positive results are likely due to the large number of different identifiers (106 to 118 mass peaks used in the Figure 1C group discrimination) as the larger the number of such identifiers, the greater the disease discriminatory capability of a platform. Results using ESI-MS were substantiated using an instrument with a different mass analyser of lower resolution; see Figure 2. These results support the hypothesis that epilepsy induces biomolecular alterations that are reflected in the peripheral blood and can play a role in identifying specific clinical groups. More specifically, these results indicate that this ESI-MS approach described has potential for monitoring and understanding epilepsy as well as aiding in therapeutic development, thus warranting further study. Of note, all of the LOOCV analyses presented in this study exhibit quite large “effect sizes” (proportional to mean differences and standard deviations of the group-specific % LOOCV classified mass peaks) in the exhibited binary comparisons. Such “effect sizes” are proportional to a Cohen’s *d* value, which is proportional to statistical power. The large Cohen’s d values in this study (Table 2) yield an estimated power of > 0.90 for these LOOCV binary comparisons and bolster the reliability of the sample sizes utilized here [27,28].

A noteworthy addition to this study is the inclusion for comparative purposes of a group of traumatic brain injury (TBI) patients without diagnosed epilepsy that were described in a previous study on TBI [14]. Others have indicated most epilepsy biomarker studies only include controls and not comparisons with other non-epileptic brain injuries and that this is a weakness in these studies [7]. Using LOOCV, this group of TBI patients (*N* = 13) segregated with the epilepsy patients when compared to controls, Figure 3A. This suggests the described above apparent physiological changes are responsible for these LOOCV serum sample separations, and the TBI condition is more related to the epilepsy condition than to the idiopathic headache condition. Importantly, a separate LOOCV analysis was able to directly discriminate the TBI patients from the epilepsy patients (Figure 3B), indicating and suggesting that different disease conditions are present.

Besides these disease group discriminations, the MS methodology employed here is able to assist in understanding biochemical mechanisms as well as identifying potential novel biomarkers and therapeutic targets. Many of the serum mass peaks analyzed in this study, for example in Figure 1C, are between approximately 500 to 1200 m/z, and likely include host tissue/organ exoprotease activities and other cell/tissue signaling activities resulting from the lower mass peptide “serome”, biomolecules [17,18]. To aid in identifying physiological differences in this complex biomolecular milieu, MS/MS structure determinations were performed. At the ionization energies employed here, intact larger proteins are not likely to be fragmented, only existing peptides and polypeptides. The identification of differentially present peptides and biochemical pathways could be helpful in understanding underlying disease mechanisms and developing novel diagnostic biomarkers and therapeutics [43].

For these purposes, a range analysis (900–1008 m/z) was conducted, revealing a prominent epilepsy phenotype with 32 of the 100 different proteins (32%) with known associations to seizure/epilepsy; see Table 3 and Table 4. A prominent epilepsy-associated protein appeared in this analysis, potassium ion-channel protein KCNQ2, whose gene plays important roles, when mutated, in the development of infancy epilepsies like benign familial neonatal convulsions (BFNC, [9]). Since epilepsy likely encompasses unknown genetic changes, the finding of KCNQ2 effects in epilepsy, indicates overlap between known gene changes in infantile epilepsy and unknown gene changes in epilepsy. Another prominent epilepsy related ion-channel protein found in this analysis is CACNA2D2, a calcium channel protein involved in small-molecule ligand interactions as well as neuronal cell death pathways [44]. These two proteins comprise two major hubs in the IPA cellular/biochemical pathway analysis overlapping a variety of epilepsies, ion-channels and synaptic transmission, and neurodegeneration and cognitive issues; see Figure 4. Other prominent hubs in the IPA using the 100 peptides/proteins listed in Table 3 and Table 4 include the blood–brain barrier (BBB), neuro-inflammation, and autoimmunity. The autoimmunity connection is of interest because it could help explain the reoccurrence and cyclical nature of seizures in epilepsy. Recent work indicates an autoimmune association with epilepsy [19]. IPA with autoimmune emphasis using the expanded list of proteins in Table S1 is exhibited in Figure S1 Encephalitis and encephalomyelitis pathways appear suggestive of viral infection origins for some epilepsy.

## 5. Conclusions

Our results demonstrate, for the patient groups analyzed, the this ESI-MS ability to platform and distinguish/monitor sera of patients having epilepsy, based on their respective serum biomolecule mass peak profiles. In addition, a set of TBI patient serum samples were segregated with epilepsy patient samples when compared with controls. Bioinformatics/systems biology analysis of MS/MS deduced peptide/protein changes associated with epilepsy, suggested cell pathways/biochemical systems affected/altered include neuro-inflammation, seizure/epilepsy, synaptic transmission, cognitive impairment, behavior, TBI, brain damage, blood–brain barrier, autoimmunity, and ion-channels.

## Figures and Tables

**Table 1:** Sociologic, demographic and clinical characteristics of participating patients with epilepsy ^a^ and control patients.

Variable	Category	Epilepsy ^a^	Control
**Sample Size**		29	17
Gender	Male	19 (66%)	7 (41%)
Age	Mean (SD)	26.9 (7.2)	34.0 (9.2)
Education	<Secondary	11 (38%)	5 (29%)
	≥Secondary	18 (62%)	12 (71%)
Consumed Pork	Yes	2 (7%)	1 (6%)
Living near a house that raised pigs ^b,c^	Yes	4 (14%)	0 (0%)
Own pigs	Yes	0 (0%)	0 (0%)
Utilize toilet to defecate? ^c^	Never	22 (76%)	16 (94%)
Cigarette smoking ^b^	Yes	4 (14%)	0 (0%)
Paan use ^b^	Yes	3 (10%)	0 (0%)
Headaches	Yes	0 (0%)	16 (94%)
Time since last seizure	≤3 months	21 (72%)	NA
	3–7 months	8 (28%)	NA
No of lifetime seizures ^a^	1	0 (0%)	NA
	2–5	9 (31%)	NA
	6–10	2 (7%)	NA
	>10	18 (62%)	NA
Type of last seizure	Generalized	24 (83%)	NA
	Partial	1 (3%)	NA
	Partial then generalized	4 (14%)	NA

^a^ unknown etiology. ^b^ These questions were only asked to male patients. ^c^ Associated with possible neurocysticercosis (a parasitic infection of *T. solium* and *T. saginata* prevalent to the area and having seizure as a clinical symptom) infection. All subjects were without edema, EITB negative, and AgELISA negative [21,22].

**Table 2:** Test metrics, patient group epilepsy vs. control comparison.

Panel I	% LOOCV Mean (SD) Group 1	% LOOCV Mean (SD) Group 2		True Positive Group 1		False Positive Group 2	True Negative Group 2	False Negative Group 1	*N*		Figure #
Group 1 vs. Group 2											
**Ion-Trap MS LOOCV Data Sets:**											
Epilepsy vs. Control	55.09%	31.81%		29/29		0/17	17/17	0/29	Epilepsy = 29; Control = 17		Figure 1C
	(4.84%)	(3.58%)		(100%)		(0%)	(100%)	(0%)			
Training: Epilepsy vs. Control	56.98%	28.60%		23/23		0/13	23/23	0/23	Epilepsy = 6; Control = 4		Figure 2B
	(4.28%)	(−4.42%)		(−100%)		(0%)	(100%)	(0%)			
Blind: Epilepsy vs. Control	47.58%	29.69%		13/13		0/16	16/16	0/13	Epilepsy = 23; Control = 13		Figure 2A
	(3.6%)	(1.18%)		(100%)		(0%)	(100%)	(0%)			
TBI vs. Epilepsy	69.73%	29.69%		13/13		0/16	16/16	0/13	TBI = 13; Epilepsy = 16		Figure 3B
	(4.48%)	(5.87%)		(100%)		(0%)	(100%)	(0%)			
**Single Quadrupole MS LOOCV Set:**											
**Epilepsy vs. Control**	47.98% (10.48%)	24.10% (9.05%)		27/29 (93.1%)		2/17 (12%)	15/17 (88.2%)	2/29 (6.9%)	Epilepsy, *N* = 29; Control, *N* = 17		Figure 2C
**Panel II**	**Sens-itivity**	**Efficiency/ [accuracy]**	**True Positive Rate**		**False Positive Rate**	**Spec-ificity**	***P*-** **value**	**Random Database *P*-value**	**Coh-en’sd**	**ROC AUC**	**Figure#**
**group 1 vs. group 2**											
**Ion-Trap MS LOOCV Data Sets**											
Epilepsy, *N* = 29 vs. Control, *N* = 17	1	1	1		0	0.97	4.56 × 10^−22^	0.108	5.46	1	Figure 1C,D
Training: Epilepsy, *N* = 23 vs. Control, *N* = 13	1	1	1		0	1	3.02 × 10^−16^	0.054	6.52	1	Figure 2A
Blind: Epilepsy, *N* = 6 vs. Control *N* = 4	1	0.9	1		0.25	0.75	5.62 × 10^−3^	na	2.07	1	Figure 2B
TBI, *N* = 13 vs. Epilepsy, *N* = 16	1	1	1		0	1	2.00 × 10^−15^	0.0012	7.66	1	Figure 3B,
**Single Quadrupole MS LOOCV Set:**											
Epilepsy, *N* = 29 vs. Control, *N* = 17	0.9	0.87	0.9		0.018	0.82	3.95 × 10^−10^	0.37	2.43	0.953	Figure 2C,D

**Table 3:** Top 50 Epilepsy over Control proteins with 2× or better sera and 1.5 or better MS/MS hits.

Symbol	Epilepsy: Control	Symbol	Epilepsy: Control	Symbol	Epilepsy: Control
	[#Sera (#Hits)]		[#Sera (#Hits)]		[#Sera (#Hits)]
**FBN3**	**6(62): 0(0)**	PEAR1 ^1,2^	4(44): 1(3)	ITGAE ^1,2,3^	3(32): 0(0)
MUC17 ^4^	6(83): 3(15)	ALPK2 ^1^	4(38): 1(10)	ATP8B2	3(30): 0(0)
SLIT2 ^1,3^	5(65): 0(0)	PKD1 ^1,4^	4(27): 1(9)	ZNF562	3(30): 0(0)
PCM1 ^1,4^	5(72): 1(6)	DACT2 ^1^	4(22): 1(9)	ADAMTSL1 ^2^	3(28): 0(0)
OTOGL	5(39): 2(10)	FBXL17	4(13): 1(3)	SLC4A4 ^1,3,4,5^	3(28): 0(0)
ARAP1 ^1,2^	4(137): 0(0)	HSPG2 ^1,2,4,5^	4(83): 2(23)	ADAMTS12 ^1,2^	3(26): 0(0)
USP19 ^1^	4(135): 0(0)	LAMA2 ^1,2,3,5^	4(67): 2(30)	CNST ^1^	3(25): 0(0)
CTCFL	4(126): 0(0)	CEP350 ^1,2^	4(33): 2(12)	KLHL4	3(25): 0(0)
SCARF1 ^2^	4(86): 0(0)	NACA ^1^	4(14): 2(9)	KCNQ2 ^1,3,4,5^	3(24): 0(0)
FAT4 ^1,3^	4(83): 0(0)	LCE1A ^2^	3(178): 0(0)	NBPF10	3(24): 0(0)
CNOT10	4(52): 0(0)	ADAM11 ^1,2,3,4,5^	3(87): 0(0)	BIN3 ^1,2,3^	3(23): 0(0)
CLINT1 ^1,2^	4(46): 0(0)	VPS13D ^1,2,3^	3(66): 0(0)	AMBN ^2^	3(22): 0(0)
CEP152	4(43): 0(0)	ADAMTS18 ^1^	3(50): 0(0)	BCL11A	3(22): 0(0)
OCA2 ^1,3,4^	4(32): 0(0)	ISM2	3(34): 0(0)	ABCA12 ^2^	3(21): 0(0)
AKAP11 ^1,4^	4(29): 0(0)	P2RX7 ^1,2,3,4,5^	3(34): 0(0)	ZNRF3 ^1^	3(21): 0(0)
LAMA3 ^2^	4(102): 1(21)	POTED	3(33): 0(0)	SLIT1 ^1^	3(20): 0(0)
EPHB2 ^1,2,3,4^	4(62): 1(12)	PCNX2 ^1,3^	3(33): 0(0)		

^1^ neurological; ^2^ immune/inflammation; ^3^ seizure/epilepsy related (shaded); ^4^ ion-channel related; ^5^ blood–brain barrier related.

**Table 4:** Top 50 Control over Epilepsy proteins with 2x or better sera and 1.5 or better MS/MS hits.

Symbol	Control: Epilepsy	Symbol	Control: Epilepsy	Symbol	Control: Epilepsy
	[#Sera (#Hits)]		[#Sera (#Hits)]		[#Sera (#Hits)]
**NOTCH2 ^1,2,4^**	**8(32): 1(3)**	RYR2 ^1,2,3,4^	4(47): 1(7)	FAT1 ^1,2^	3(51): 0(0)
NOTCH2NL ^1^	7(49): 1(2)	TNXA ^1^	4(41): 1(23)	NF1 ^1,2,3,4^	3(44): 0(0)
ZFP1	7(190): 3(21)	RECK ^1,2,3,5^	4(34): 1(7)	PSD3 ^1,4^	3(44): 0(0)
PKHD1 ^2^	5(41): 0(0)	ELF2 ^1^	4(28): 1(3)	USP28 ^1,2^	3(42): 0(0)
DST ^1,2^	5(192): 1(9)	THSD7A ^1^	4(20): 1(3)	KMT5B ^1,2,3^	3(38): 0(0)
VWF ^1,2,3,4,5^	5(182): 1(3)	C16orf96	4(12): 1(3)	CEP295	3(35): 0(0)
MEGF8	5(52): 1(9)	MEGF11 ^1^	4(56): 2(21)	ZMYM4 ^1^	3(35): 0(0)
ATP10A ^1,3,4^	5(40): 1(4)	ASS1 ^1,2,3^	4(40): 2(21)	LAMB4 ^2^	3(34): 0(0)
BS ^1,2,3,4^	5(20): 1(5)	FGFR3 ^1,3^	4(37): 2(25)	HDC ^1,2,3,4,5^	3(31): 0(0)
CACNA2D2 ^1,3,4^	4(222): 0(0)	KIF9 ^2^	3(150): 0(0)	FOXA1 ^1,2,4^	3(29): 0(0)
APC ^1,2,3^	4(57): 0(0)	NRAP	3(120): 0(0)	ADAMTSL3 ^1,2^	3(28): 0(0)
ZNF300 ^1,2^	4(36): 0(0)	ATP7B ^1,2,3,4,5^	3(98): 0(0)	NAV3 ^1^	3(28): 0(0)
CSMD1 ^1,2,3^	4(32): 0(0)	ABL2 ^1,2,3^	3(67): 0(0)	GNPTAB ^1,2^	3(27): 0(0)
PCSK1 ^1^	4(90): 1(11)	ADGRG4	3(67): 0(0)	ASTN2 ^1,3,4^	3(25): 0(0)
MT-CO1 ^1,3^	4(86): 1(15)	RAPGEF6 ^1^	3(58): 0(0)	ADNP ^1,2,3,4^	3(23): 0(0)
RNF213 ^1,2,3^	4(78): 1(15)	MAGED4 ^1^	3(52): 0(0)	DMAC1	3(23): 0(0)
CRIM1 ^1^	4(76): 1(9)	CHFR ^1,2^	3(51): 0(0)		

^1^ neurological; ^2^ immune/inflammation; ^3^ seizure/epilepsy related (shaded); ^4^ ion-channel related; ^5^ blood–brain barrier related.

## Supplementary Materials

The following are available online at https://www.mdpi.com/2076-3425/10/8/504/s1. Figure S1: IPA m/z Range MS/MS Serum Data Analysis for Epilepsy Patients vs. Controls using top 182 Peptide/Proteins from Table S1 with autoimmune emphasis. Table S1: Top 182 proteins found in MS/MS analysis using 2× sera no. filter and 1.5× MS/MS “hit” filter.
