# Supplementary material for: Distinguishing and Biochemical Phenotype Analysis of Epilepsy Patients Using a Novel Serum Profiling Platform
Source: Brain Sci. 2020 Jul 31;10(8):504. doi: 10.3390/brainsci10080504 (PMC7464346; doi:10.3390/brainsci10080504)
Supplement: Supplementary file 1 [file brainsci-10-00504-s001.zip › S1 Figure.docx]

**S1 Figure: IPA m/Z Range MS/MS Serum Data Analysis for Epilepsy Patients vs Controls using top 182 Peptide/Proteins from Table S1 with autoimmune emphasis.**


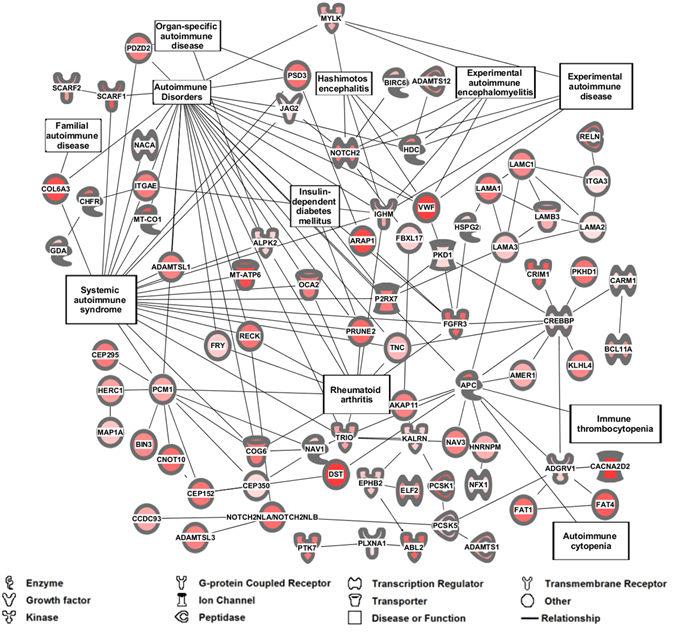


**S1 Figure.** IPA m/Z Range MS/MS Serum Data Analysis for Epilepsy Patients vs Controls using top 182 Peptide/Proteins from STable 1. Ingenuity Pathway Analysis (IPA), Qiagen, Inc.) of the 100 peptides/proteins exhibited in STable 1 having a 2x difference in positive sera number between epilepsy and controls, and a 1.5x difference in MS/MS “hit” [single peptide identification] ratio between the two groups. The protein function legend is at the bottom of the figure..
