# Supplementary material for: Distinguishing and Biochemical Phenotype Analysis of Epilepsy Patients Using a Novel Serum Profiling Platform
Source: Brain Sci. 2020 Jul 31;10(8):504. doi: 10.3390/brainsci10080504 (PMC7464346; doi:10.3390/brainsci10080504)
Supplement: Supplementary file 1 [file brainsci-10-00504-s001.zip › S1_Table_.docx]

| **S1 Table: Top 182 proteins found in MS/MS analysis using 2x sera no. filter and 1.5x MS/MS “hit” filter** | | | | | | | | |
| --- | --- | --- | --- | --- | --- | --- | --- | --- |
| I | **Proteins expressed in EUE over Control (Panel I)** | | | | | | | |
| Rank | **Symbol** | **EUE : Control**  **[#Sera (#hits)]** | **Rank** | **Symbol** | **EUE : Control**  **[#Sera (#hits)]** | **Rank** | **Symbol** | **EUE : Control**  **[#Sera (#hits)]** |
| 1 | FBN3 | 6(62) : 0(0) | 31 | ISM2 | 3(34) : 0(0) | 61 | AMER1 | 3(8) : 0(0) |
| 2 | MUC17 | 6(83) : 3(15) | 32 | P2RX7 | 3(34) : 0(0) | 62 | BIRC6 | 3(66) : 1(9) |
| 3 | SLIT2 | 5(65) : 0(0) | 33 | POTED | 3(33) : 0(0) | 63 | NAV1 | 3(65) : 1(15) |
| 4 | PCM1 | 5(72) : 1(6) | 34 | PCNX2 | 3(33) : 0(0) | 64 | TNC | 3(62) : 1(8) |
| 5 | OTOGL | 5(39) : 2(10) | 35 | ITGAE | 3(32) : 0(0) | 65 | IARS | 3(56) : 1(3) |
| 6 | ARAP1 | 4(137) : 0(0) | 36 | ATP8B2 | 3(30) : 0(0) | 66 | NFX1 | 3(54) : 1(19) |
| 7 | USP19 | 4(135) : 0(0) | 37 | ZNF562 | 3(30) : 0(0) | 67 | JAG2 | 3(48) : 1(18) |
| 8 | CTCFL | 4(126) : 0(0) | 38 | ADAMTSL1 | 3(28) : 0(0) | 68 | FRY | 3(45) : 1(11) |
| 9 | SCARF1 | 4(86) : 0(0) | 39 | SLC4A4 | 3(28) : 0(0) | 69 | LAMB3 | 3(43) : 1(4) |
| 10 | FAT4 | 4(83) : 0(0) | 40 | ADAMTS12 | 3(26) : 0(0) | 70 | ADGRV1 | 3(42) : 1(11) |
| 11 | CNOT10 | 4(52) : 0(0) | 41 | CNST | 3(25) : 0(0) | 71 | LINC02280 | 3(40) : 1(3) |
| 12 | CLINT1 | 4(46) : 0(0) | 42 | KLHL4 | 3(25) : 0(0) | 72 | KALRN | 3(39) : 1(10) |
| 13 | CEP152 | 4(43) : 0(0) | 43 | KCNQ2 | 3(24) : 0(0) | 73 | GDA | 3(33) : 1(6) |
| 14 | OCA2 | 4(32) : 0(0) | 44 | NBPF10 | 3(24) : 0(0) | 74 | HNRNPM | 3(31) : 1(3) |
| 15 | AKAP11 | 4(29) : 0(0) | 45 | BIN3 | 3(23) : 0(0) | 75 | MYLK | 3(31) : 1(2) |
| 16 | LAMA3 | 4(102) : 1(21) | 46 | AMBN | 3(22) : 0(0) | 76 | SSH1 | 3(30) : 1(9) |
| 17 | EPHB2 | 4(62) : 1(12) | 47 | BCL11A | 3(22) : 0(0) | 77 | CREBBP | 3(29) : 1(9) |
| 18 | PEAR1 | 4(44) : 1(3) | 48 | ABCA12 | 3(21) : 0(0) | 78 | SIMC1 | 3(27) : 1(3) |
| 19 | ALPK2 | 4(38) : 1(10) | 49 | ZNRF3 | 3(21) : 0(0) | 79 | KIF6 | 3(26) : 1(3) |
| 20 | PKD1 | 4(27) : 1(9) | 50 | SLIT1 | 3(20) : 0(0) | 80 | GPR176 | 3(26) : 1(1) |
| 21 | DACT2 | 4(22) : 1(9) | 51 | HDAC5 | 3(17) : 0(0) | 81 | PLXNA1 | 3(21) : 1(8) |
| 22 | FBXL17 | 4(13) : 1(3) | 52 | HTR5A | 3(17) : 0(0) | 82 | DNER | 3(21) : 1(7) |
| 23 | HSPG2 | 4(83) : 2(23) | 53 | TCF20 | 3(17) : 0(0) | 83 | PLXNA3 | 3(20) : 1(7) |
| 24 | LAMA2 | 4(67) : 2(30) | 54 | TNN | 3(15) : 0(0) | 84 | CAMTA1 | 3(19) : 1(6) |
| 25 | CEP350 | 4(33) : 2(12) | 55 | CACNG8 | 3(14) : 0(0) | 85 | ITGA3 | 3(18) : 1(7) |
| 26 | NACA | 4(14) : 2(9) | 56 | UNC5A | 3(14) : 0(0) | 86 | PCSK5 | 3(18) : 1(3) |
| 27 | LCE1A | 3(178) : 0(0) | 57 | HERC1 | 3(11) : 0(0) | 87 | CARM1 | 3(17) : 1(5) |
| 28 | ADAM11 | 3(87) : 0(0) | 58 | CCDC93 | 3(9) : 0(0) | 88 | CCDC141 | 3(16) : 1(7) |
| 29 | VPS13D | 3(66) : 0(0) | 59 | IGHM | 3(9) : 0(0) | 89 | MAP1A | 3(13) : 1(2) |
| 30 | ADAMTS18 | 3(50) : 0(0) | 60 | SLC33A1 | 3(9) : 0(0) |  |  |  |
|  |  |  |  |  |  |  |  |  |
| **II** | **Proteins expressed in Control over EUE** | | | | | | | |
| **Rank** | **Symbol** | **Control : EUE [#Sera (#hits)]** |  | **Symbol** | **Control: EUE [#Sera (#hits)]** | **Rank** | **Symbol** | **Control: EUE [#Sera (#hits)]** |
| 1 | NOTCH2 | 8(32) : 1(3) | 32 | RAPGEF6 | 3(58) : 0(0) | 63 | TRIO | 3(11) : 0(0) |
| 2 | NOTCH2NL | 7(49) : 1(2) | 33 | MAGED4 | 3(52) : 0(0) | 64 | DCAF15 | 3(9) : 0(0) |
| 3 | ZFP1 | 7(190) : 3(21) | 34 | CHFR | 3(51) : 0(0) | 65 | MUM1 | 3(9) : 0(0) |
| 4 | PKHD1 | 5(41) : 0(0) | 35 | FAT1 | 3(51) : 0(0) | 66 | TRO | 3(244) : 1(14) |
| 5 | DST | 5(192) : 1(9) | 36 | NF1 | 3(44) : 0(0) | 67 | MT-ATP6 | 3(114) : 1(8) |
| 6 | VWF | 5(182) : 1(3) | 37 | PSD3 | 3(44) : 0(0) | 68 | COL6A3 | 3(98) : 1(23) |
| 7 | MEGF8 | 5(52) : 1(9) | 38 | USP28 | 3(42) : 0(0) | 69 | EYA1 | 3(66) : 1(11) |
| 8 | ATP10A | 5(40) : 1(4) | 39 | KMT5B | 3(38) : 0(0) | 70 | RNF17 | 3(64) : 1(11) |
| 9 | BSN | 5(20) : 1(5) | 40 | CEP295 | 3(35) : 0(0) | 71 | ZAN | 3(59) : 1(5) |
| 10 | CACNA2D2 | 4(222) : 0(0) | 41 | ZMYM4 | 3(35) : 0(0) | 72 | ZNF41 | 3(47) : 1(7) |
| 11 | APC | 4(57) : 0(0) | 42 | LAMB4 | 3(34) : 0(0) | 73 | PRUNE2 | 3(47) : 1(9) |
| 12 | ZNF300 | 4(36) : 0(0) | 43 | HDC | 3(31) : 0(0) | 74 | ZNF76 | 3(46) : 1(3) |
| 13 | CSMD1 | 4(32) : 0(0) | 44 | FOXA1 | 3(29) : 0(0) | 75 | CFAP54 | 3(41) : 1(21) |
| 14 | PCSK1 | 4(90) : 1(11) | 45 | ADAMTSL3 | 3(28) : 0(0) | 76 | C6 | 3(39) : 1(13) |
| 15 | MT-CO1 | 4(86) : 1(15) | 46 | NAV3 | 3(28) : 0(0) | 77 | RELN | 3(39) : 1(13) |
| 16 | RNF213 | 4(78) : 1(15) | 47 | GNPTAB | 3(27) : 0(0) | 78 | PTK7 | 3(32) : 1(3) |
| 17 | CRIM1 | 4(76) : 1(9) | 48 | ASTN2 | 3(25) : 0(0) | 79 | ZNF681 | 3(32) : 1(20) |
| 18 | RYR2 | 4(47) : 1(7) | 49 | ADNP | 3(23) : 0(0) | 80 | LAMA1 | 3(26) : 1(7) |
| 19 | TNXA | 4(41) : 1(23) | 50 | DMAC1 | 3(23) : 0(0) | 81 | KMT2C | 3(23) : 1(5) |
| 20 | RECK | 4(34) : 1(7) | 51 | CASKIN1 | 3(21) : 0(0) | 82 | PIGZ | 3(23) : 1(5) |
| 21 | ELF2 | 4(28) : 1(3) | 52 | HIVEP2 | 3(20) : 0(0) | 83 | VWCE | 3(22) : 1(7) |
| 22 | THSD7A | 4(20) : 1(3) | 53 | ARMCX4 | 3(18) : 0(0) | 84 | ZNF462 | 3(22) : 1(14) |
| 23 | C16orf96 | 4(12) : 1(3) | 54 | TRANK1 | 3(17) : 0(0) | 85 | COG6 | 3(21) : 1(7) |
| 24 | MEGF11 | 4(56) : 2(21) | 55 | ABCA3 | 3(16) : 0(0) | 86 | CFAP47 | 3(21) : 1(9) |
| 25 | ASS1 | 4(40) : 2(21) | 56 | LRRC37A3 | 3(14) : 0(0) | 87 | NTNG2 | 3(18) : 1(7) |
| 26 | FGFR3 | 4(37) : 2(25) | 57 | ZNF536 | 3(14) : 0(0) | 88 | PDZD2 | 3(18) : 1(12) |
| 27 | KIF9 | 3(150) : 0(0) | 58 | SCARF2 | 3(13) : 0(0) | 89 | LAMC1 | 3(16) : 1(9) |
| 28 | NRAP | 3(120) : 0(0) | 59 | KIR2DL5A | 3(12) : 0(0) | 90 | RP1L1 | 3(15) : 1(5) |
| 29 | ATP7B | 3(98) : 0(0) | 60 | DUOXA1 | 3(11) : 0(0) | 91 | USH2A | 3(13) : 1(4) |
| 30 | ABL2 | 3(67) : 0(0) | 61 | ERV3-1 | 3(11) : 0(0) | 92 | PARP4 | 3(13) : 1(5) |
| 31 | ADGRG4 | 3(67) : 0(0) | 62 | TMEM259 | 3(11) : 0(0) | 93 | MEGF9 | 3(12) : 1(3) |
